# Evaluation of mHealth Apps for Diverse, Low-Income Patient Populations: Framework Development and Application Study

**DOI:** 10.2196/29922

**Published:** 2022-02-11

**Authors:** Shreya Sharma, Katherine Gergen Barnett, John (Jack) Maypole, Rebecca Grochow Mishuris

**Affiliations:** 1 Boston University School of Medicine Boston, MA United States; 2 Beth Israel Deaconess Medical Center Boston, MA United States; 3 Department of Family Medicine Boston Medical Center Boston, MA United States; 4 Department of Family Medicine Boston University School of Medicine Boston, MA United States; 5 Department of Pediatrics Boston Medical Center Boston, CA United States; 6 Department of Pediatrics Boston University School of Medicine Boston, MA United States; 7 Department of Medicine Boston Medical Center Boston, MA United States; 8 Department of Medicine Boston University School of Medicine Boston, MA United States

**Keywords:** mobile health application, apps, mobile health, diverse, low-income, mHealth, framework, chronic disease, condition, smoking cessation, diabetes, medication adherence, safety net hospital, personal, self-management, usability test

## Abstract

**Background:**

The use of mobile technology or smartphones has grown exponentially in the United States, allowing more individuals than ever internet access. This access has been especially critical to households earning less than US $30,000, the majority of whom indicate that smartphones are their main source of internet access. The increasing ubiquity of smartphones and virtual care promises to offset some of the health disparities that cut through the United States. However, disparities cannot be addressed if the medical information offered though smartphones is not accessible or reliable.

**Objective:**

This study seeks to create a framework to review the strengths and weaknesses of mobile Health (mHealth) apps for diverse, low-income populations.

**Methods:**

Focusing on smoking cessation, diabetes management, and medication adherence as models of disease management, we describe the process for selecting, evaluating, and obtaining patient feedback on mHealth apps.

**Results:**

The top 2 scoring apps in each category were QuitNow! and Smoke Free-Quit Smoking Now for smoking cessation, Glucosio and MyNetDiary for diabetes management, and Medisafe and MyMeds for medication adherence.

**Conclusions:**

We believe that this framework will prove useful for future mHealth app development, and clinicians and patient advisory groups in connecting culturally, educationally, and socioeconomically appropriate mHealth apps with low-income, diverse communities and thus work to bridge health disparities.

## Introduction

The use of mobile technology has grown exponentially in the United States, and the COVID-19 pandemic both enforced the need for internet connectivity and laid bare disparities in access. Currently, up to 97% of Americans own a cell phone, including 97% of those with a household income under US $30,000 [1]. However, 27% of households earning under US $30,000 rely on smartphones for internet access, compared to only 6% of households earning greater than US $100,000 [2]. During the COVID-19 pandemic, 43% of individuals used their cell phones to manage mental health and well-being, 41% to access health care, and 40% to keep fit and exercise [3]. While there still remains a large socioeconomic digital divide, the increasing ownership of ever-evolving smartphones and their functionality may partially offset this disparity [4,5].

The mobile health (mHealth) app market has flourished as consumers, health technology companies, and biomedical researchers have recognized mobile apps as a potential vehicle to lower barriers to accessing preventive medicine and promoting healthy behaviors. This extends to lower-income populations as well [6]. Currently, there are a total of 48,608 mHealth apps available for download in the Apple App Store [7]. The mHealth app industry is forecasted to be worth US $151 billion by 2025 [8,9]. However, little is known about efficacy of these apps, particularly across diverse consumer (and patient) populations.

The COVID-19 pandemic has illuminated the long prevalent health disparities among lower-income populations who experience higher rates of chronic disease such as diabetes and hypertension [10]. Mobile apps could, in theory, address some of these disparities. Indeed, mobile health apps have been proven, in many instances, to impact lifestyle changes and health outcomes positively [11,12]. There are available mobile health apps that promote healthy behaviors (such as exercise or smoking cessation) and support chronic disease management (such as monitoring blood sugar levels and enhancing medication adherence). Patients in diverse, low-income communities have shown more interest than white, high-income communities in the use of mHealth apps, particularly for chronic disease and overall health management [13-15]. This presents an area of opportunity for software developers and health care providers to reduce health inequities. However, the vast majority of mHealth apps do not cater to the needs of lower-income populations, as they have been shown to be difficult to navigate for individuals who may have limited health, digital, or written literacy [11,16-20]. Additionally, there is no universally accepted framework to assess the functionality and usability of mHealth apps, which may further disproportionately impact diverse, low-income populations [6,12].

This study seeks to create a framework for the evaluation of mHealth apps’ accessibility for diverse, low-income populations. We developed and tested a rubric (a guide listing specific criteria for grading or scoring) of domains (features of mHealth apps with a common purpose) to measure the functionality and usability of several mHealth apps for patients of an urban safety net institution. Focusing on smoking cessation, diabetes management, and medication adherence as models of disease management, we describe the process of selection of domains of mHealth apps, development of the rubric, and scoring of various mHealth apps. We envision that this framework will prove useful for clinicians, care teams, patient advisory groups, and developers (who seek to design apps with equitable reach and greater impact on health outcomes) in connecting culturally, educationally, and socioeconomically appropriate mHealth apps with communities historically overlooked by this rapidly evolving area of health care.

## Methods

### Domain Selection

In June 2018, we searched web-based databases (PubMed and Embase) to identify articles related to the evaluation of the usability of mobile apps for health and wellness. Studies from this literature review were assessed to create a list of domains for rating mHealth apps relevant to diverse, low-income populations. Search criteria included “smartphone application underserved community,” “usability of commercially available applications for diverse patients,” “mHealth apps usability testing underserved,” “mheatlh app underserved,” “mobile health phone applications for diverse populations,” on PubMed and “mobile application'/exp OR 'mobile application' OR 'mobile phone'/exp OR 'mobile phone,” “‘mHealth under-served' OR (('mhealth'/exp OR mhealth) AND unders-erved)” on Embase.

An extensive literature review indicated important domains of mHealth apps for our target population, including the following: language, literacy, graphics, multimedia, usability, patient-centeredness, data entry mode, data exportability, cost, evidence based content, platform, extent to which the platform was up to date, connectivity, Americans with Disabilities Act (ADA) accessibility, privacy, social network, cultural sensitivity (incorporating diversity in language, graphics, and data), messaging or reminder capability, and benchmarking (comparing a user’s performance to the performance of others on the app). These domains were grouped into larger categories, including the following: Usability (graphics, multimedia, usability, and ADA accessibility), Population focus (language, literacy, patient-centeredness, cost, social network, cultural sensitivity, benchmarking, and messaging or reminder capability), Technology (data entry mode, data exportability, platform, connectivity requirement, and privacy), and Clinical Impact (evidence-based content and extent to which the platform was up to date).

Each domain was then weighted in terms of importance to the target population by independent coders (RGM, KGB, and JM). These coders are all primary care physicians at an urban safety net hospital (in General Internal Medicine, Family Medicine, and Pediatrics, each with 10-25 years of experience at this institution) and care for overlapping members of these communities by age. They were asked to rate each domain on a scale of 1 to 5 (1=“not important to be included” and 5=“must be included”). Then, a reviewer (SS) adjudicated these weights and averaged the “weight” of each domain respectively. Finally, these weights were directionally confirmed with the hospital-based patient and family advocacy committee.

### App Selection

To simulate how patients would access recommendations for mHealth apps, we used the most common search engine, Google, to search “Top Ten Mobile Health Apps for Diabetes” and “Top Ten Mobile Health Apps for Smoking Cessation.” The first search result referred to articles in a popular health website “Healthline,” which provided the top 10 mobile health apps related to each topic [21,22]. The website rated these apps on the basis of quality, reliability, reviews, and community nominations. As no such list existed for medication adherence focused apps, these apps were chosen from the list recommended by a previous study [23]. We chose apps in these 3 areas because they represent different aspects of medical care delivery: preventative care (smoking cessation), chronic disease management (diabetes management), and general therapeutic intervention (medication adherence).

### App Scoring

Ten apps in smoking cessation, diabetes management, and medication adherence were identified and rated by a coder (SS) in each domain (language, literacy, graphics, multimedia, usability, patient-centered, data entry mode, data exportability, cost, evidence-based content, platform, extent the platform was up to date, connectivity, ADA accessibility, privacy, social network, cultural sensitivity, messaging or reminder capability, and benchmarking) with a score of 0 to 3. A score of 0 was assigned if the specific domain was not applicable, and a score of 3 was assigned for the highest applicability (eg, language—available in 3 or more languages; Table 1). Domains were defined on the basis of current research in each respective domain. For example, for “Social Network,” higher points were assigned for “competition” than for “social support” because previous studies have shown that social comparison was more important for physical activity [24]. As another example, the definition for “Usability” was based on the design of the app and the ability to navigate it easily. We specifically did not use the industry standard assessment (ie, System Usability Scale) owing to concerns about the applicability for diverse, low–socioeconomic status communities. Lastly, we decided whether apps were “evidence based” if they cited specific research or if the theory underlying their apps had existing evidence (ie, gasification and social networks).

The domain score was multiplied by the domain weight (of importance to the target population) to produce a weighted final score for that domain for the app in question. The weighted scores across all domains were added to assign each app a final overall score of usefulness and applicability for diverse, low-income populations.

**Table 1:** Domain definitions and scoring.

Domain		Points			
		0	1	2	3
**Population focus**					
	Language	Not applicable/not offered by app/not a function of this app	Only available in English	Available in English + 1 language	Multiple (>2) languages
	Literacy	Not applicable/not offered by app/not a function of this app	Complex language, medical jargon	some patient-friendly language, some medical jargon	patient-friendly language at or below 5th grade reading level
	Patient-centeredness	Not applicable/not offered by app/not a function of this app	Does not allow patient-entered data/provides no tailored content	Provides moderate tailored content	Provides actionable content based on patient-entered data
	Social network	Not applicable/not offered by app/not a function of this app	Provides a forum for people to share information/discuss different topics	Provides a platform for competition between users	Incorporates a forum and also a “competition” between users
	Benchmarking	Not applicable/not offered by app/not a function of this app	Provides no sense of patient status among peers	Provides a benchmark against others	Allows patients to define peer groups for benchmarking
	Cultural sensitivity	N/A^a^	No attempt at cultural diversity	Some attempt at cultural diversity	Cultural diversity definitely represented
	Cost	Not applicable/not offered by app/not a function of this app	Paid app	Can pay for add-on	Free app
	Messages or reminders	Not applicable/not offered by app/not a function of this app	Has messages, but not tailored to the user	App has motivational messages tailored to the user/reminders pop-up, but only if the user is on the app	App has option for you to “turn on notification” to receive reminders, messages, and notifications tailored specifically to the user
**Usability**					
	Graphics	Not applicable/not offered by app/not a function of this app	Only text	Text>graphics	Graphics clearly explain the text
	Multimedia	Not applicable/not offered by app/not a function of this app	No use of audio or video	Uses audio or video	Uses audio and video
	Usability	Not applicable/not offered by app/not a function of this app	Complex app navigation, small icons, multiple screens, not immediately obvious how to use	Small icons, multiple screens, simpler app navigation but still would require instruction	Large icons, few screens, easy to navigate without instruction/mobile literacy
	Americans with Disabilities Act accessibility	Not applicable/not offered by app/not a function of this app	No accommodation for visual or hearing impairment	Accommodation for visual or hearing impairment	Accommodation for both visual and hearing impairment
**Technology**					
	Data entry mode	Not applicable/not offered by app/not a function of this app	Manual entry	Manual entry or integrated with information from the phone/other devices	Integrated with external devices or electronic health records
	Data exportability	Not applicable/not offered by app/not a function of this app	Does not allow data export	Allows export of data to print or email or message	Integrated with external services or electronic health records
	Connectivity requirement	Not applicable/not offered by app/not a function of this app	Requires constant Wi-Fi/cellular connection	Some functions useful without connection	Fully functional without connection/only requires connection to integrate with external devices/system
	Platform	Not applicable/not offered by app/not a function of this app	Only iOS	Only android	iOS and android
	Privacy	Not applicable/not offered by app/not a function of this app	No privacy statement	N/A	Privacy statement exists
**Clinical impact**					
	Evidence-based content	Not applicable/not offered by app/not a function of this app	No clear identification of evidence for content	Some content has an evidence base	Entire app content is evidence-based
	Up to date	Not applicable/not offered by app/not a function of this app	No indication of last update date/ updated >18 months ago	Updated within the last 12-18 months	Updated within last 6-12 months

^a^N/A: not applicable.

### Patient Advisory Board

In July 2019, after the mHealth apps were chosen and scored, we presented the top 3 apps in each health category to a patient advisory board at a safety net hospital in Boston, Massachusetts. The group consisted of 4 participants on the day of presentation. They were Caucasian women between 35 and 75 years of age from the catchment area of the safety net institution. They were asked to give their overall thoughts on our research idea and the functionality of mHealth apps they considered important to manage their health on a daily basis.

## Results

### Domain Scoring

Domains of usability and functionality for mHealth apps were assigned weighted scores depending on the importance the clinical experts gave to those categories for diverse, low-income communities. These were directionally confirmed with a patient advisory board. Domains of greatest importance were identified through this process (Table 2). Literacy was found to be the highest scoring domain by clinical experts, with all raters assigning a score of 5 out of 5. Language, usability, cost, evidence based content, and cultural sensitivity had an average weight of 4.5 out of 5. Graphics was rated 4 out of 5. Social media connectivity and timing of the app’s latest update were assigned a score of 3.5 out of 5. Multimedia, data exportability, patient centeredness, benchmarking, and messages or reminders were all rated an average of 3 out of 5. ADA accessibility was rated 2.5 out of 5. Data entry and privacy domains were weighted 2 out of 5, being the least important for the specific patient population. Raters were within 1 point of each other on all domains, with 8 out of 19 domain weightings being identical. The higher-level categories into which domains can be grouped also differentiated in terms of importance, with clinical impact and population focus being most highly weighted: Clinical Impact (average weight across component domains 4), Population Focus (average weight across component domains 3.88), Usability (average weight across component domains 3.5), and Technology (average weight across component domains 2.8).

**Table 2:** Domains of greatest importance.

Domain		Average final weight
**Population focus**		
	Literacy	5
	Language	4.5
	Cultural sensitivity	4.5
	Cost	4.5
	Graphics	4
	Social network	3.5
	Patient-centeredness	3
	Benchmarking	3
**Usability**		
	Usability	4.5
	Multimedia	3
	Messages or reminders	3
	Americans with Disabilities Act accessibility	2.5
**Technology**		
	Platform	4
	Data exportability	3
	Connectivity requirement	3
	Data entry mode	2
	Privacy	2
**Clinical impact**		
	Evidence-based content	4.5
	Up to date	3.5

### App Scoring

When using the weighted scoring methodology to rate mHealth apps across smoking cessation, diabetes management, and medication adherence, the framework was able to sufficiently distinguish among 10 apps within each category (Table 3). Weighted scores ranged from 108.5 to 153 for smoking cessation apps, 119 to 147 for diabetes management apps, and 113 to 137.5 for medication adherence apps (Table 4). The top 2 scoring apps in each category were QuitNow! (156/201), Smoke Free-Quit Smoking Now (149.5/201), Glucosio (147/201), MyNetDiary (146/201), Medisafe (137.5/201), and MyMeds (126/201) (Multimedia Appendix 1).

**Table 3:** Weighted scores for each top mobile app.

	Smoking cessation		Diabetes management			Medication adherence	
	QuitNow!	Smoke Free- Quit Smoking Now	MyNetDiary	Glucosio	Medisafe		My Meds
Rating on app store (out of 5)	4.6	4.8	4.6	N/A^a^	4.7		N/A
Ratings, n	317,000	181,000	107,000	N/A	138,000		N/A
Language (4.5)	13.5	13.5	4.5	13.5	3		1
Literacy (5)	15	15	15	15	15		15
Graphics (4)	8	12	12	12	12		12
Multimedia (3)	3	6	6	3	3		3
Usability (4.5)	13.5	13.5	9	13.5	13.5		13.5
Patient- centeredness (3)	6	9	9	3	9		3
Data entry mode (2)	2	2	4	2	6		2
Data exportability (3)	6	6	9	6	6		3
Cost (4.5)	9	9	6	13.5	9		13.5
Evidence-based content (4.5)	13.5	13.5	13.5	4.5	0		0
Platform (4)	12	12	12	12	12		12
Up to date (3.5)	10.5	10.5	10.5	10.5	10.5		10.5
Connectivity requirement (3)	6	6	6	9	9		9
Americans with Disabilities Act accessibility (2.5)	2.5	2.5	2.5	2.5	2.5		2.5
Privacy (2)	6	6	6	6	6		6
Benchmarking (3)	6	3	3	3	3		3
Social support (3.5)	3.5	0	3.5	0	0		3.5
Cultural sensitivity (4.5)	4.5	4.5	13.5	9	9		4.5
Messages (3)	9	9	9	9	9		9

^a^N/A: not applicable.

**Table 4:** Weighted scores of top mobile apps.

App name			Cumulative final score		Range of final scores across all 10 apps evaluated
**Smoking cessation**					108.5-153
	Smoke Free-Quit Smoking Now	153			
	QuitNow!	146.5			
**Diabetes management**					122-147
	Glucosio	147			
	MyNetDiary	146			
**Medication adherence**					113-137.5
	Medisafe	137.5			
	My Meds	126			

## Discussion

### Principal Findings

mHealth apps have the potential to improve individuals’ management of chronic diseases and to extend the reach of the health care provider visit. Considering the increasing predominance of smart device use to access the internet among diverse, low-income communities, mHealth apps hold enormous potential for impact on health and could help bridge health equity divides. To date, however, studies have shown that more research is needed to rate the practical functionality of these mobile apps, specifically for this target population [25,26]. By developing a framework to rate the usefulness of mHealth applications for low income, diverse, patient populations, and showing its effectiveness to differentiate between apps, we aimed to provide a way to curate mHealth apps for better access and engagement among diverse populations.

Existing frameworks such as the Mobile App Rating Scale (MARS) include ratings for domains grouped into categories with broad population applicability, including engagement, functionality, aesthetics, subjective quality, and information. As identified by researchers who created the MARS system, this grading system is agnostic to the needs of specific populations and may not be indicative of the usefulness of apps for specific groups. As a result, it is difficult to apply the grading criteria of MARS to diverse, low-income patient populations [26]. The evaluation mechanism described in this study incorporates domains that are applicable for general consumption as contained in MARS and highlighted by Anderson et al [27] and others as important to engagement (“evidence based content,” “privacy,” “up to date,” “patient centered,” “benchmarking,” and “social network”), but expands beyond MARS specifically for diverse, low-income patient populations [26-29].

In contrast to the more generalized domains, our research led us to evaluate a greater number of criteria with population-specific focus. The domains expanded upon those in the MARS criteria to include: language, literacy, cultural sensitivity, data entry, data exportability, multimedia, ADA accessibility, cost, platform, and message or reminder function. As identified by prior studies, multimedia availability, such as videos, helped increase mobile app engagement, particularly for individuals with low literacy levels [16,17,19,20,30,31]. Messages or a reminder function helped app users stay motivated in their plan, manage medications, and organize personal health information [29,32-36]. Cost was a domain included in our scoring framework because some past studies showed increased engagement if patients paid for the app. However, there is a question as to whether this would be an undue barrier for patients with low income [27]. Importantly and consistent with our hypothesis, language, literacy, and cultural sensitivity were found to be some of the most important qualities for an app to have to be relevant and useful for diverse, low-income populations, according to both our clinician and patient reviewers [14,16,18-20,29,37-40].

The highest rated mHealth apps in our sample shared notable, similar qualities, regardless of what chronic illness or health state they were developed to manage. Most apps placed importance on offering multiple languages, being written for lower literacy levels, and incorporating graphics; these scored high on usability. However, apps such as MyNetDiary, for diabetes management, which were more difficult to navigate, did offer additional resources to learn about navigating through the app, such as an instructional video. Additionally, privacy, evidence-based content, data entry, data exportability, and patient-centered content were present in most (but not all) of the chosen apps. Benchmarking (comparing a user’s performance to the performance of others on the app), social support networks, and multimedia use scores were not robust in any of the chosen apps. ADA accessibility and cultural sensitivity (incorporating diversity in language, graphics, and data) were also lacking. Based on these results, the apps rated most highly in accordance with our framework conveyed information in a manner that a more diverse user base would be able to effectively engage with. However, as a whole, the mobile apps did not focus any specific attention to engaging a diverse population. For example, although many apps were inclusive by providing multiple language options, they lacked content that was tailored to a culturally diverse population. Overall, although these apps were the best of what is currently on the market, they are far from being ideal for our target population.

The patient advisory board was both a versatile and key feature of our research effort providing both a patient perspective on the mHealth apps and on the domains included in the framework we developed. Thus, we gained insight to patients’ perspectives and were able to compare their priorities in engaging with these apps to those of health care providers in the same system. Domains including language, literacy, evidence-based content, cultural sensitivity, and providing up-to-date information were ranked highly by both groups. However, some domains such as graphics, social network, and benchmarking were not as important to patients as they were thought to be by providers. In contrast to our qualitative findings, prior studies have shown that apps with simple interfaces that favor graphics over text tend to be more usable with lower literacy populations [19,37]. Additionally, social media functionality has also been found to reduce barriers to sharing information and learning from others [32,41,42]. Therefore, while other studies have shown these domains to be important to engagement, our patients did not rank them highly in terms of importance to usability. These differences could be explained by the small sample size and demographics of our patient advisory group.

### Limitations

There were several limitations to this work, which can be used as learning points for future research. First, in selecting which mHealth apps to evaluate we used a common website which had its own subjective way of choosing which apps were the “best.” However, this approach to finding the “top 10” apps on a website likely replicates the way in which patients would search for and find mHealth apps to download and use. The mHealth app market is in a constant state of flux and growth, and with it, the “top 10.” Second, some of the mHealth apps initially identified for evaluation were not available for download. Churn in the mHealth market may impact engagement with some mHealth apps. However, this better reflected which mHealth apps would be accessible to our patients. Third, as this was a small study with no independent funding, we only had one reviewer grade the mHealth apps across each domain. Though individual user subjectivity is inherently part of the process of evaluating mHealth apps, this process may result in some subjectivity to our analysis of the individual apps. Furthermore, our patient advisory board was not necessarily representative of the diverse patient population of the hospital. While they themselves were patients at the safety net institution, they were all White women aged 35-75 years. As a result, their opinions may not be generalizable and could be subject to conscious and unconscious biases. While this limitation is significant, we also found it important to have some patient perspective and validation rather than none at all. Finally, it is important to acknowledge our mHealth rubric and research was conducted before the COVID-19 pandemic, and much of mHealth has evolved since then. Our reliance on technology is more than ever, and we may be more dependent on mobile app technologies. However, the nature of this research should withstand the evolution of the mHealth app market, and even seek to improve upon it as it can be applied to make this growing field even more accessible for a diverse user base.

### Conclusions

We adapted a framework for evaluating mHealth apps for diverse, low-income populations from the MARS model and created modified domains to characterize critical features as identified by patients and clinical experts who care for these patient populations. Although these app domains are rated separately, it is important to remember that often these different domains work synergistically. Some domains may increase user engagement and retention while others are focused on increasing access, inclusivity, or privacy. Multiple languages remain a worthy goal, but we contend mHealth app developers should also consider incorporating culturally sensitive and specific information such as recipes, videos, and motivational tools.

This novel framework is invaluable as it can be applied to evaluate individual mHealth apps in the context of therapeutic interventions, as well as by app developers to identify those domains important for engagement of diverse, low-income populations. However, right now our app ranks relative performance. To establish specific scoring thresholds and determine cut-off ratings for apps we need to apply this rating framework to a large number of apps. The plan would be to accomplish this during a follow-up study. For now, we believe distinguishing relative performance is important so developer can use an iterative approach to design apps. They can use scores as a performance indicator as they improve or refine existing apps. If developers are able to report app performance, it may be a way to filter apps and create inter-app competition to improve performance.

### Future Prospects

In the future, the study team plans to incorporate the selected apps in smoking cessation, diabetes management, and medication adherence into our clinical processes as “prescriptions” for patients. We will be able to track these “prescriptions,” assess patient engagement, and determine impact on health outcomes. In addition, we hope to continue to use our framework to find, scale, and spread mHealth apps that will be most useful for our target population in other areas of health including exercise and mental health. By using this novel framework to identify mHealth apps for recommendation and for future mHealth app development, health care providers, policy makers and developers alike may be able to better incorporate this burgeoning technology into both clinical practice and patient homes for greater impact on health outcomes for all patients, further narrowing digital and health outcome divides.

## Appendix

Multimedia Appendix 1 Domain Definitions and Scoring.

## Abbreviations

ADA: Americans with Disabilities Act
mHealth: mobile health
