# Supplementary material for: Evaluation of mHealth Apps for Diverse, Low-Income Patient Populations: Framework Development and Application Study
Source: JMIR Form Res. 2022 Feb 11;6(2):e29922. doi: 10.2196/29922 (PMC8881782; doi:10.2196/29922)
Supplement: Multimedia Appendix 1 [file formative_v6i2e29922_app1.docx]

**Multimedia Appendix 1. Domain Definitions and Scoring.**

| **Weighted Scores for Each Top Mobile Application** | | | | | | | | | | | | | | | | | | | | |
| --- | --- | --- | --- | --- | --- | --- | --- | --- | --- | --- | --- | --- | --- | --- | --- | --- | --- | --- | --- | --- |
| Smoking Cessation | | | | | | | | | | | | | | | | | | | | |
| **Mobile Application Name** | | LiveStrong MyQuit Coach | | Quit Smoking: Cessation Nation | | Craving to Quit | | QuitNow! | | Quit Smoking with Andrew Johnson | | Smoke Free- Quit Smoking Now | | Kwit | Butt Out | | Get Rich or Die Smoking | | Smoke Free- Quit Smoking Slowly | |
| **Rating (x/5)** | | 4.4 | | 4.5 | | 4.5 | | 4.6 | | n/a | | 4.8 | | 4.6 | 4.5 | | 4.6 | | 4.4 | |
| **Number of Ratings** | | 1.3K | | 6.6K | | 20 | | 3.17K | | n/a | | 18.1K | | 525 | 21 | | 12.7K | | 7.3K | |
| **Language (4.5)** | | 4.5 | | 4.5 | | 4.5 | | 13.5 | | 4.5 | | 13.5 | | 13.5 | 13.5 | | 13.5 | | 4.5 | |
| **Literacy (5)** | | 15 | | 15 | | 15 | | 15 | | 15 | | 15 | | 15 | 15 | | 15 | | 15 | |
| **Graphics (4)** | | 12 | | 8 | | 12 | | 8 | | 0 | | 12 | | 12 | 4 | | 8 | | 8 | |
| **Multimedia (3)** | | 3 | | 3 | | 9 | | 3 | | 6 | | 6 | | 3 | 9 | | 6 | | 3 | |
| **Usability (4.5)** | | 13.5 | | 9 | | 9 | | 13.5 | | 13.5 | | 13.5 | | 13.5 | 9 | | 9 | | 9 | |
| **Patient- centered (3)** | | 6 | | 6 | | 6 | | 6 | | 3 | | 9 | | 6 | 3 | | 9 | | 6 | |
| **Data Entry Mode (2)** | | 2 | | 2 | | 4 | | 2 | | 0 | | 2 | | 2 | 4 | | 2 | | 2 | |
| **Data Exportability (3)** | | 3 | | 3 | | 3 | | 6 | | 3 | | 6 | | 6 | 6 | | 3 | | 3 | |
| **Cost (4.5)** | | 9 | | 9 | | 9 | | 9 | | 4.5 | | 9 | | 9 | 13.5 | | 9 | | 9 | |
| **Evidence Based Content (4.5)** | | 13.5 | | 9 | | 13.5 | | 13.5 | | 13.5 | | 13.5 | | 13.5 | 4.5 | | 13.5 | | 9 | |
| **Platform (4)** | | 4 | | 8 | | 4 | | 12 | | 12 | | 12 | | 12 | 12 | | 8 | | 8 | |
| **Up To Date (3.5)** | | 10.5 | | 3.5 | | 10.5 | | 10.5 | | 3.5 | | 10.5 | | 10.5 | 7 | | 3.5 | | 7 | |
| **Connectivity Requirement (3)** | | 9 | | 6 | | 6 | | 6 | | 9 | | 6 | | 9 | 3 | | 6 | | 6 | |
| **ADA-accessibility (2.5)** | | 2.5 | | 5 | | 2.5 | | 2.5 | | 2.5 | | 2.5 | | 2.5 | 2.5 | | 2.5 | | 2.5 | |
| **Privacy (2)** | | 6 | | 4 | | 6 | | 6 | | 2 | | 6 | | 6 | 6 | | 6 | | 6 | |
| **Benchmarking (3)** | | 3 | | 6 | | 3 | | 6 | | 3 | | 3 | | 3 | 3 | | 3 | | 3 | |
| **Social Support (3.5)** | | 3.5 | | 3.5 | | 3.5 | | 3.5 | | 0 | | 0 | | 0 | 3.5 | | 3.5 | | 3.5 | |
| **Cultural Sensitivity (4.5)** | | 4.5 | | 4.5 | | 4.5 | | 4.5 | | 4.5 | | 4.5 | | 4.5 | 4.5 | | 4.5 | | 4.5 | |
| **Messages (3)** | | 6 | | 6 | | 9 | | 9 | | 9 | | 9 | | 6 | 6 | | 3 | | 9 | |
|  | |  | |  | |  | |  | |  | |  | |  |  | |  | |  | |
|  | |  | |  | |  | |  | |  | |  | |  |  | |  | |  | |
| **Weighted Scores for Each Top Mobile Application** | | | | | | | | | | | | | | | | | | | | |
| **Medication Adherence** | | | | | | | | | | | | | | | | | | | | |
| **Mobile Application Name** | Medisafe | | CareZone | | My Pillbox* | | Dosecast | | My Meds | | MyMedManager* | | Pill Reminder | | | MediWare* | | Medicine List* | | My heart, My life* |
| **Rating (x/5)** | 4.7 | | 4.6 | |  | | 3.7 | | n/a | |  | | 4.7 | | |  | |  | |  |
| **Number of Ratings** | 13.8K | | 8.3K | |  | | 47 | |  | |  | | 3.06K | | |  | |  | |  |
| **Language (4.5)** | 3 | | 1 | |  | | 3 | | 1 | |  | | 3 | | | 1 | |  | |  |
| **Literacy (5)** | 15 | | 5 | |  | | 15 | | 15 | |  | | 15 | | |  | |  | |  |
| **Graphics (4)** | 12 | | 12 | |  | | 4 | | 12 | |  | | 8 | | |  | |  | |  |
| **Multimedia (3)** | 3 | | 3 | |  | | 3 | | 3 | |  | | 3 | | |  | |  | |  |
| **Usability (4.5)** | 13.5 | | 9 | |  | | 13.5 | | 13.5 | |  | | 13.5 | | |  | |  | |  |
| **Patient- centered (3)** | 9 | | 3 | |  | | 3 | | 3 | |  | | 6 | | |  | |  | |  |
| **Data Entry Mode (2)** | 6 | | 2 | |  | | 2 | | 2 | |  | | 2 | | |  | |  | |  |
| **Data Exportability (3)** | 6 | | 6 | |  | | 6 | | 3 | |  | | 6 | | |  | |  | |  |
| **Cost (4.5)** | 9 | | 13.5 | |  | | 9 | | 13.5 | |  | | 9 | | |  | |  | |  |
| **Evidence Based Content (4.5)** | 0 | | 0 | |  | | 0 | | 0 | |  | | 0 | | |  | |  | |  |
| **Platform (4)** | 12 | | 12 | |  | | 12 | | 12 | |  | | 12 | | |  | |  | |  |
| **Up To Date (3.5)** | 10.5 | | 10.5 | |  | | 10.5 | | 10.5 | |  | | 10.5 | | |  | |  | |  |
| **Connectivity Requirement (3)** | 9 | | 9 | |  | | 9 | | 9 | |  | | 9 | | |  | |  | |  |
| **ADA-accessibility (2.5)** | 2.5 | | 2.5 | |  | | 2.5 | | 2.5 | |  | | 2.5 | | |  | |  | |  |
| **Privacy (2)** | 6 | | 6 | |  | | 6 | | 6 | |  | | 6 | | |  | |  | |  |
| **Benchmarking (3)** | 3 | | 3 | |  | | 3 | | 3 | |  | | 3 | | |  | |  | |  |
| **Social Support (3.5)** | 0 | | 3.5 | |  | | 3.5 | | 3.5 | |  | | 0 | | |  | |  | |  |
| **Cultural Sensitivity (4.5)** | 9 | | 9 | |  | | 4.5 | | 4.5 | |  | | 4.5 | | |  | |  | |  |
| **Messages (3)** | 9 | | 9 | |  | | 9 | | 9 | |  | | 0 | | |  | |  | |  |
|  |  | |  | |  | |  | |  | |  | |  | | | * = unable to access app in the US or app no longer existed in app store | |  | |  |
| **Weighted Scores for Each Top Mobile Application** | | | | | | | | | | | | | | | | | | | | |
| **Diabetes Management** | | | | | | | | | | | | | | | | | | | | |
| **Mobile Application Name** | Fooducate | | Glooko | | Health2Sync | | MyNetDiary | | Diabetes Tracker by MyNetDiary** | | MySugr: Diabetes Tracker Log | | BG Monitor* | | | BeatO | | Glucosio | | Diabetes Connect |
| **Rating (x/5)** | 4.7 | | 3 | | 4.6 | | 4.6 | | 4.5 | | 4.6 | | 4.5 | | | n/a | | n/a | | 4.5 |
| **Number of Ratings** | 21K | | 38 | | 47 | | 1.07K | | 232 | | 1.38K | | 654 | | | n/a | | n/a | | 37 |
| **Language (4.5)** | 4.5 | | 4.5 | | 13.5 | | 4.5 | | 4.5 | | 13.5 | | 4.5 | | | 4.5 | | 13.5 | | 13.5 |
| **Literacy (5)** | 15 | | 15 | | 15 | | 15 | |  | | 15 | |  | | | 15 | | 15 | | 15 |
| **Graphics (4)** | 4 | | 12 | | 12 | | 12 | |  | | 12 | |  | | | 8 | | 12 | | 12 |
| **Multimedia (3)** | 6 | | 3 | | 3 | | 6 | |  | | 3 | |  | | | 6 | | 3 | | 3 |
| **Usability (4.5)** | 9 | | 9 | | 9 | | 9 | |  | | 9 | |  | | | 4.5 | | 13.5 | | 13.5 |
| **Patient- centered (3)** | 6 | | 3 | | 3 | | 9 | |  | | 3 | |  | | | 3 | | 3 | | 3 |
| **Data Entry Mode (2)** | 4 | | 4 | | 4 | | 4 | |  | | 4 | |  | | | 2 | | 2 | | 2 |
| **Data Exportability (3)** | 6 | | 6 | | 6 | | 9 | |  | | 6 | | 6 | | | 6 | | 6 | | 6 |
| **Cost (4.5)** | 9 | | 13.5 | | 9 | | 6 | | 4.5 (9.99) | | 9 | | 9 | | | 13.5 | | 13.5 | | 9 |
| **Evidence Based Content (4.5)** | 13.5 | | 4.5 | | 4.5 | | 13.5 | |  | | 4.5 | | 13.5 | | | 4.5 | | 4.5 | | 4.5 |
| **Platform (4)** | 12 | | 12 | | 12 | | 12 | | 12 | | 12 | | 8 | | | 12 | | 12 | | 12 |
| **Up To Date (3.5)** | 10.5 | | 10.5 | | 10.5 | | 10.5 | | 10.5 | | 10.5 | | 9 | | | 10.5 | | 10.5 | | 10.5 |
| **Connectivity Requirement (3)** | 6 | | 6 | | 6 | | 6 | |  | | 6 | | 6 | | | 3 | | 9 | | 9 |
| **ADA-accessibility (2.5)** | 2.5 | | 2.5 | | 2.5 | | 2.5 | |  | | 2.5 | | 2.5 | | | 2.5 | | 2.5 | | 2.5 |
| **Privacy (2)** | 6 | | 6 | | 6 | | 6 | | 6 | | 6 | | 6 | | | 6 | | 6 | | 6 |
| **Benchmarking (3)** | 3 | | 3 | | 3 | | 3 | |  | | 3 | |  | | | 3 | | 3 | | 3 |
| **Social Support (3.5)** | 3.5 | | 0 | | 3.5 | | 3.5 | |  | | 0 | |  | | | 0 | | 0 | | 0 |
| **Cultural Sensitivity (4.5)** | 13.5 | | 4.5 | | 9 | | 13.5 | |  | | 9 | |  | | | 9 | | 9 | | 9 |
| **Messages (3)** | 9 | | 0 | | 0 | | 9 | |  | | 9 | |  | | | 9 | | 9 | | 9 |

| * = unable to access |
| --- |
| ** too expensive, did not evaluate |
